# Nonequilibrium Synthesis of Glycolamide (NH_2_COCH_2_OH), a Precursor to Amino Acids, on Interstellar Nanoparticles

**DOI:** 10.1021/acscentsci.5c01856

**Published:** 2025-12-24

**Authors:** Alexandre Bergantini, Jia Wang, Ivan Antonov, Evgenia A. Batrakova, Sergey O. Tuchin, Ralf I. Kaiser

**Affiliations:** ∥ W. M. Keck Research Laboratory in Astrochemistry, 3949 University of Hawaii at Manoa, Honolulu, Hawaii 96822, United States; ‡ Department of Chemistry, 3949 University of Hawaii at Manoa, Honolulu, Hawaii 96822, United States; § 65055 Samara National Research University, Samara 443086 Russia

## Abstract

Complex organic molecules (COMs) are thought to form in cold interstellar environments, yet the chemical routes to key prebiotic precursors remain poorly understood. Glycine, the simplest amino acid, has not been detected in the interstellar medium so far, prompting interest in its structural isomer glycolamide, recently observed toward Sgr B2. Here, we provide the first experimental evidence that glycolamide forms efficiently at cryogenic temperatures via a barrierless carbon–carbon bond coupling between two carbon-centered carbamoyl (ĊONH_2_) and hydroxymethyl (ĊH_2_OH) radicals, generated from formamide and methanol in astrophysical ice analogues exposed to cosmic-ray proxies. This radical–radical pathway proceeds within the ices of ice-coated grains over typical dense cloud lifetimes (∼10^6^ years), thus establishing a nonequilibrium mechanism to glycine isomers of astrobiological relevance. Once formed, glycolamide can act as a versatile precursor to amino acids and sugars thereby contributing to the molecular inventory inherited by nascent exoplanetary systems such as Fomalhaut along with Alpha Lyrae and our own.

## Graphical abstract

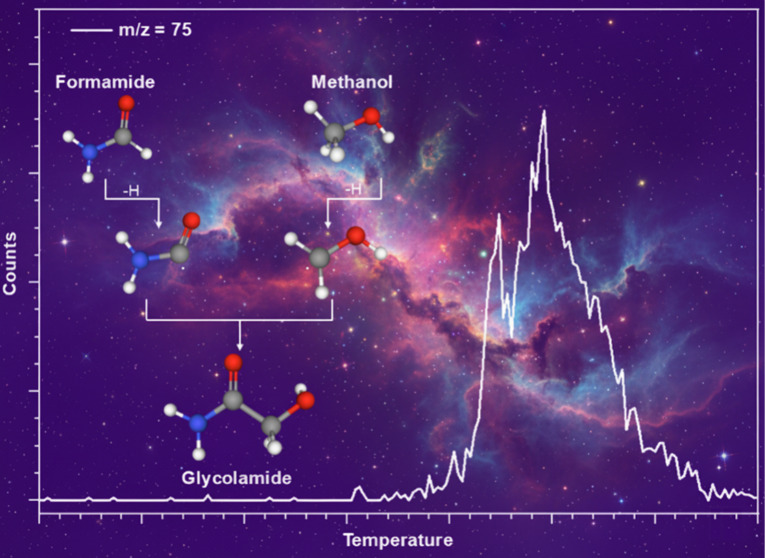

## Introduction

An understanding of the origin of life on early Earth requires a thorough investigation of the fundamental formation mechanisms of biorelevant, complex organic molecules (COMs)–carbon-based molecules with six or more atoms defined by the astronomy community.^1^ These organics perform fundamental structural (cell membranes),^2^ enzymatic (Krebs cycle),^3^ and regulatory (glycolysis)^4^ functions–topics that have garnered significant interest across the fields of astrochemistry, organic synthesis, physical chemistry, and astrobiology.^5–8^ COMs represent a dominant fraction of close to 50% of some 330 molecules detected in deep space so far.^9^ These COMs emerge from intricate chemical processes commencing in cold molecular clouds and extend into star forming regions along with protoplanetary disks.^8,10^ However, the mechanisms underlying the synthesis and transformation of critical biorelevant precursors to complex biotic compounds such as glycine (NH_2_CH_2_COOH, **1**), the simplest amino acid and a fundamental building block of polypeptides, remains poorly understood, despite the exceptional body of work already available in the literature.^11–15^ Two primary hypotheses have been proposed to explain the origin of COMs on the early Earth: an *in situ* synthesis on the planetary surface such as in hydrothermal vents^16^ or a delivery via exogenous sources like comets and meteorites.^17^ Impact models by Chyba et al.^17^ suggest that an extraterrestrial delivery of prebiotic organic compounds to the early Earth contributed up to 3 orders of magnitude more matter than could have been synthesized through endogenous processes on Earth including Miller-Urey-type reactions, volcanic activity, and hydrothermal systems.^18^ Consequently, an advancement of our fundamental understanding of the chemical composition of interstellar molecular clouds is essential for reconstructing the processes that led to the emergence of life on early Earth and potentially on exoplanets in habitable zones such as Kepler-452b,^19^ TRAPPIST-1,^20^ and HD 141399.^21^ In this sense, the search for amino acids in the interstellar medium (ISM) has been ongoing for nearly half a century.^22–28^

Although glycine (**1**) has been identified in chondritic meteorites such as Murchison, Murray, and Allende,^29,30^ comets like 81P/Wild and 67P/Churyumov–Gerasimenko,^31,32^ and very recently on the carbonaceous asteroid Ryugu^33^ with abundances of up to 6.1 μg/g, **1** has not yet been detected in the ISM. The absence of **1** in the ISM represents a significant unresolved puzzle in astrochemistry.^34^ One explanation is that **1** is either absent from the ISM or present at abundances below current observational detection limits.^26^ To address this “missing glycine” issue, astronomers have turned their focus to a key structural isomer of glycine: glycolamide (NH_2_COCH_2_OH, **2**) (Figure 1); this isomer has been recently detected toward the star forming region Sgr B2 of the Galactic center.^26^ Glycolamide (**2**) may serve as a precursor to key prebiotic molecules: the carbon–oxygen bond cleavage of **2** yields acetamide (CH_3_CONH_2_, **3**) and ethanolamine (HOCH_2_CH_2_NH_2_, **4**), a critical molecular building block that functions as a headgroup in phosphatidylethanolamine, a component of eukaryotic and bacterial membranes.^35^ Oxidation of **4**, driven by ultraviolet (UV) photons or galactic cosmic rays (GCRs), leads to the formation of **1**. Glycolamide (**2**) can also be converted to glycolic acid (HOCH_2_COOH, **5**) via nucleophilic substitution; **5** acts as a critical branching point: substitution of the hydroxyl group with an amino group regenerates glycine, whereas the addition of a methyl radical yields lactic acid (CH_3_CH­(OH)­COOH, **6**), a key metabolic intermediate leading to sugar acids. The hydroxyl/amino group exchange in **6** produces the amino acid alanine (C_3_H_7_NO_2_, **7**), while reduction of compound **6** results in lactaldehyde (C_3_H_6_O_2_, **8**)–a key intermediate to interstellar sugars.^36^ Furthermore, hydroxyl loss, caused by GCRs or UV photons, and subsequent reaction with deprotonated glycine results in l-asparagine (C_4_H_8_N_2_O_3_, **9**), an α-amino acid present in the biosynthesis of proteins.^37^ Consequently, glycolamide (**2**) is of significant interest in the context of prebiotic chemistry, as it may serve as a central molecular precursor to essential biomolecules including amino acids, sugars, and sugar acids. However, the formation pathways of **2** in deep space have remained elusive.

**Figure 1:**
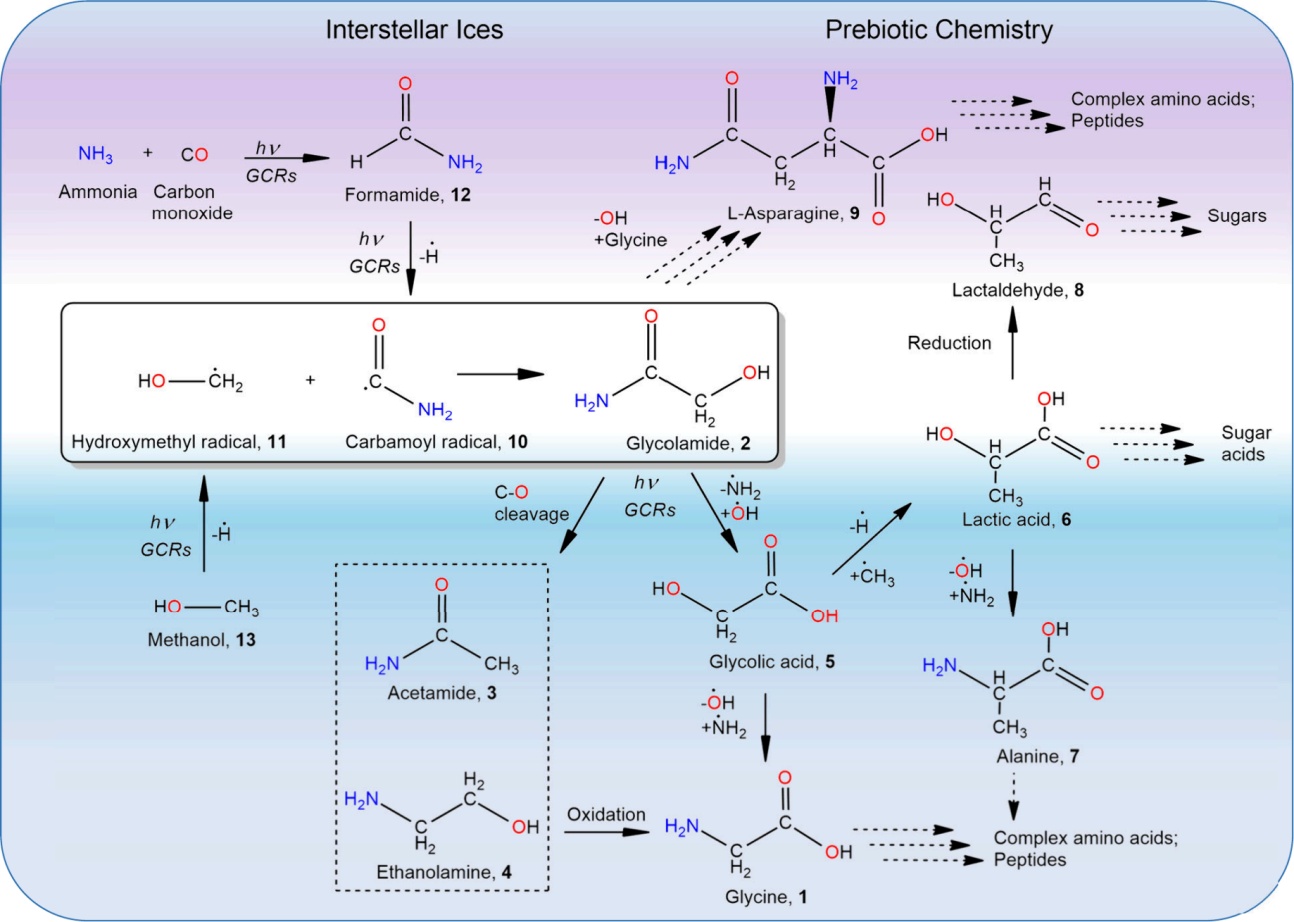
Formation of glycolamide (**2**) in interstellar ices and its role as a precursor to amino acids. Glycolamide is synthesized in simulated interstellar ice containing formamide (**12**) and methanol (**13**). These ices undergo energetic processing by galactic cosmic rays (GCRs) leading to carbon–carbon bond formation via radical–radical recombination between the hydroxymethyl radical (ĊH_2_OH, **11**) and the carbamoyl radical (ĊONH_2_, **10**). **2** can subsequently undergo molecular mass growth processes to glycine (NH_2_CH_2_COOH, **1**) through oxidation of ethanolamine (**4**) or amino/hydroxyl exchange in glycolic acid (HOCH_2_COOH, **5**). The proposed reaction network further suggests the formation of biologically relevant molecules, including lactic acid (CH_3_CH­(OH)­COOH, **6**) and lactaldehyde (CH_3_CH­(OH)­CHO, **8**) driven by the interaction of energetic radiation with ice-coated interstellar grains.

Here, we present a combined experimental and theoretical investigation demonstrating, for the first time, the formation of **2** on the surface and within the bulk of model interstellar nanoparticles, which are nanometer-sized (1–100 nm) carbonaceous, silicate, or metallic particles present in the ISM. Compound **2** is generated through a nonequilibrium synthesis process in which energetic electrons, analogous to the secondary particles produced along the tracks of GCRs, deposit energy into the ice. This energy input drives a range of physicochemical transformations, including localized heating, sputtering, compaction, and extensive radical formation. In our experiments, the primary radicals originate from H-abstraction in formamide and methanol molecules. The reaction of interest then proceeds via a barrierless radical–radical recombination involving carbon–carbon coupling between the carbamoyl (ĊONH_2_, **10**) and hydroxymethyl (ĊH_2_OH, **11**) radicals. Formamide (**12**) and methanol (**13**), two molecular species abundantly detected in interstellar molecular clouds^38,39^ (Figures 1 and 2). This reaction occurs under low temperature conditions of 5 to 10 K, mimicking the chemistry on ice coated interstellar grains exposed to proxies of GCRs over time scales of some 10^6^ years^40^ (Figure 2). These organics may enter the gas phase via shock sputtering^41^ or thermal desorption during cloud collapse and transformation into star forming regions via sublimation to be eventually incorporated into protoplanetary disks. This chemistry can be preserved on icy objects formed in the midplane region of the protostellar cloud.^42^ In our Solar system, this process resulted in the formation of Trans Neptunian Objects (TNOs)–some 1000 planetary bodies beyond the orbit of Neptune. Subsequent dynamical evolution perturbs TNOs into the inner solar system orbits, where they are relabeled as comets, thus enabling delivery of prebiotic material via impacts.^43^ This scenario is supported by the compositional similarity between cometary volatiles (1P/Haley,^44^ C/1995 O1 Hale-Bopp^45^) and protostellar molecular clouds,^46^ like TMC 1 ^47^ and RCrA.^48^ Therefore, an exogenous delivery of complex organic molecules–particularly prebiotic compounds such as **2**–likely played a pivotal role in the origin of life not only on Earth but also in extrasolar planetary systems such as Fomalhaut and Alpha Lyrae, where Kuiper Belt analog structures have been detected.^49,50^

**Figure 2:**
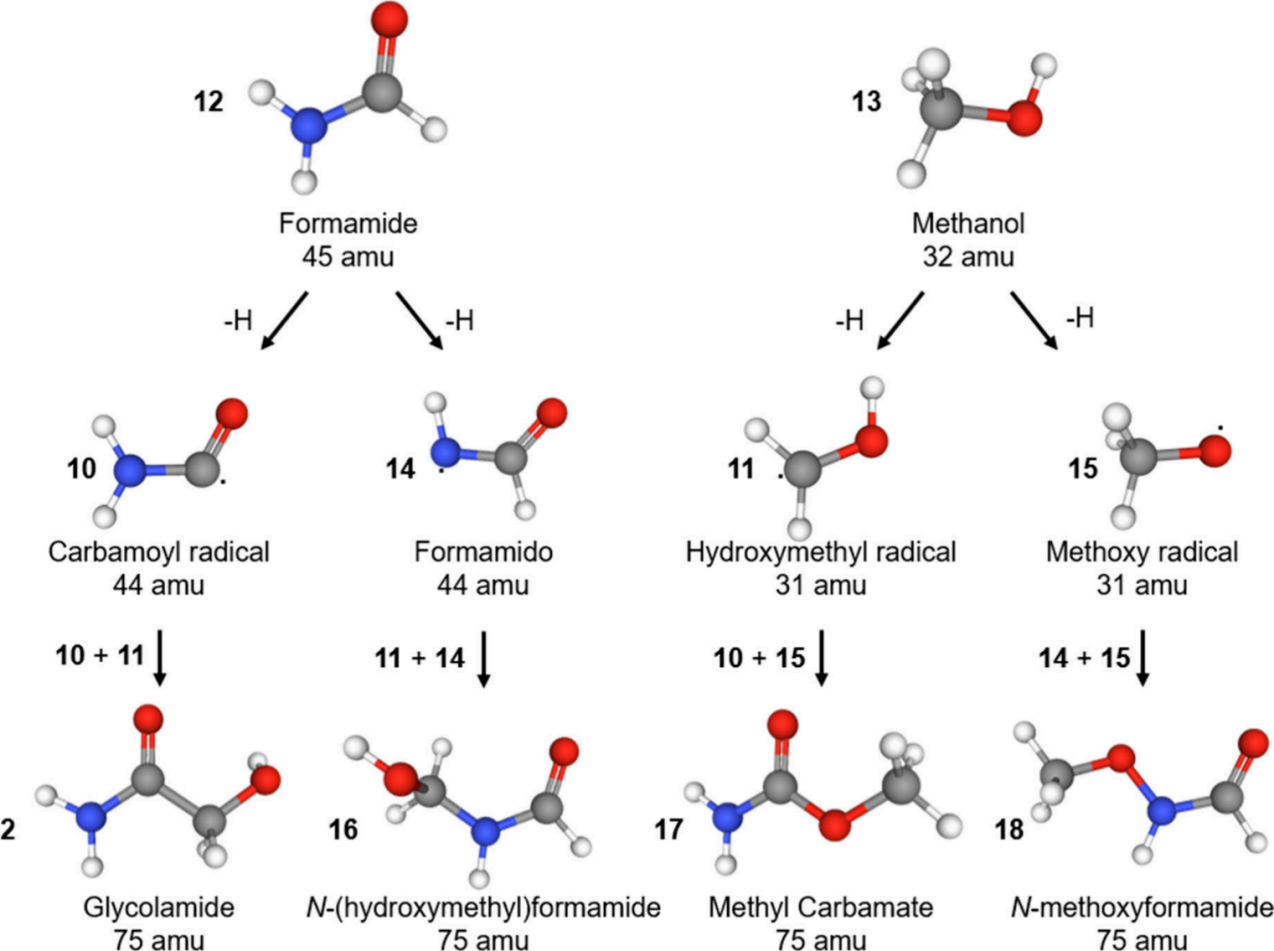
Reaction scheme leading to first generation products in low dose exposures of formamide–methanol ices at 5 K. Carbon atoms are colored gray, hydrogens are colored white, oxygens are colored red, and nitrogens are colored blue.

## Results and Discussion

### Infrared Spectroscopy

Fourier transform infrared (FTIR) spectroscopy was used to monitor the HCONH_2_–CH_3_OH (≈1/1 ratio) ices and the isotopically labeled system (HCOND_2_–CH_3_OD) before, during, and after the irradiation with proxies of GCRs in the form of energetic electrons. The IR spectra can be seen in Figure S1. Prior to the irradiation, the infrared absorptions can be linked to the vibrational modes of the reactants, formamide and methanol, such as the C–O stretching mode at 1044 cm^–1^ (ν_8_), the ν_4_, ν_5_, ν_6_, and ν_10_ modes located between 1372 cm^–1^ and 1538 cm^–1^ (CH_3_ asymmetric, symmetric, asymmetric bends and O–H bend, respectively), and the C–H symmetric stretching mode at 2834 cm^–1^ of methanol. The ν_1_ mode of methanol, which is characterized by a wide band at the 3000 cm^–1^ and 3600 cm^–1^ range, is blended with the ν_1_ and ν_2_ modes of formamide (NH_2_ antisymmetric and symmetric stretching, respectively). The NH_2_ waging (689 cm^–1^) and twisting (634 cm^–1^) modes of formamide (ω NH_2_ and 2τ NH_2_, respectively) are also merged with the O–H out-of-plane bending mode of methanol (693 cm^–1^). Moreover, the key infrared absorption features that distinguish formamide include C = O stretching (ν_4_) at 1687 cm^–1^, C–H in-plane scissoring (ν_6_) at 1388 cm^–1^, and C–N stretching (ν_7_) at 1329 cm^–1^. The infrared vibrational modes and their respective assignments detected before and after the irradiation in our experiments can be seen in Table 1. As a result of the low irradiation dose applied, only three new IR bands emerged at 2135 cm^–1^, 2167 cm^–1^, and 2267 cm^–1^. The 2135 cm^–1^ band is assigned to carbon monoxide (CO), while the 2167 cm^–1^ band corresponds to the asymmetric stretch mode of the cyanate ion (OCN^–^).^51,52^ The third band at 2267 cm^–1^ is consistent with the N=C=O asymmetric stretch commonly assigned to isocyanic acid (HNCO).^52,53^ No infrared absorptions corresponding to C_2_H_5_NO_2_ isomers were detected in the experiments, as the irradiation dose is too low to produce enough of these compounds for FTIR detection. This highlights the need for a more sensitive technique, such as PI-ReTOF-MS, for their observation.

**Table 1:** Infrared Absorption Features Recorded before and after Irradiation of Formamide–Methanol Ices at 5 K

Assignment	Position (cm^–1^)/(μm)	Reference
Before Irradiation		
ν_1_ asym. NH_2_ stretch (HCONH_2_)	3318/3.01	^52^
ν_1_ OH stretch (CH_3_OH)	3672–3019/2.72–3.31	^54^
ν_2_ sym. NH_2_ stretch (HCONH_2_)	3164/3.16	^52^
asym. CH_3_ stretch (CH_3_OH)	2957/3.38	^55^
ν CH stretch (HCONH_2_)	2904/3.44	^56^
ν_3_ sym. str. CH_3_ stretch (CH_3_OH)	2827/3.53	^54^
ν_3_ CH stretch (HCONH_2_)	2881/3.47	^52^
2δ CH bend overtone (HCONH_2_)	2802/3.56	^56^
Combination (CH_3_OH)	2531/3.95	^54^
ν_1_ CO stretch (CO_2_)	2341/4.27	^52^
Overtone of CH_3_ rock (CH_3_OH)	2235/4.47	^55^
Overtone of C–O stretch (CH_3_OH)	2036/4.91	^55^
ν_4_ CO stretch (HCONH_2_)	1685/5.93	^52^
ν_5_ in plane NH_2_ sciss. (HCONH_2_)	1644/6.08	^52^
ν_4_ CH_3_ sym. a. bend (CH_3_OH)	1461/6.84	^54^
ν_6_ in plane CH scissoring (HCONH_2_)	1385/7.22	^52^
ν_7_ CN stretch (HCONH_2_)	1324/7.55	^56^
CH_3_ rock (CH_3_OH)	1129/8.85	^57^
ρ NH_2_ rock (HCONH_2_)	1114/8.97	^56^
ν_8_ CO stretch (CH_3_OH)	1031/9.69	^54^
ω NH_2_ wag (HCONH_2_)	723/13.83	^56^
OH out-of-plane bend (CH_3_OH)	693/14.43	^55^
2τ NH_2_ twist (HCONH_2_)	610/16.39	^56^
After Irradiation		
N=C=O asym. stretch (HNCO)	2267/4.41	^52^
N=C=O sym. stretch (OCN^–^)	2167/4.61	^52^
C≡O stretch (CO)	2135/4.68	^52^

### Photoionization Reflectron Time-of-Flight Mass Spectrometry

The formation of glycolamide (**2**) and its structural isomers was investigated by using tunable photoionization reflectron time-of-flight mass spectrometry (PI-ReTOF-MS), a technique that enables isomer-selective detection based on adiabatic ionization energy (IE) and characteristic sublimation behavior during temperature-programmed desorption (TPD), linked with isotopic substitution experiments. Upon irradiation of formamide–methanol ices at 5 K, reaction intermediates and products can form in the bulk of the ice. As the temperature is gradually increased, parent and daughter species sublime into the gas phase, where they are photoionized by vacuum ultraviolet (VUV) photons and thus detected by time-of-flight mass spectrometry. Only molecules with IEs lower than the photon energy of the VUV photons used in each experiment can be photoionized and subsequently detected, allowing for a selective identification of structural isomers. Specific isomer assignments are further supported by their unique desorption temperature profiles and diagnostic mass-to-charge (*m*/*z*) shifts observed in isotopically labeled experiments. The PI-ReTOF-MS data obtained during the TPD of the ices are compiled in Figure 3. Note that, as experimental adiabatic IEs for the relevant C_2_H_5_NO_2_ isomers were not available in the literature, theoretical calculations had to be performed to obtain these values. Neutral and cationic structures of the first-generation products (see Figure 2), glycolamide (**2**), *N*-(*hydroxymethyl*)­formamide (**16**), methylcarbamate (NH_2_COOCH_3_, **17**), and *N-methoxyformamide* (CH_3_ONHCOH, **18**), were fully optimized, and their electronic energies were computed using the composite method Complete Basis Set–Quadratic Configuration Interaction–3-parameter^58^ (CBS–QB3) with B3LYP geometry optimization, carried out using the GAUSSIAN 09 software package^59^ (Table S1). Optimized geometries were generated for all relevant conformers associated with each backbone isomer (**2**, **16**, **17**, and **18**, Figure 2). Based on the calculated IEs, three vacuum ultraviolet (VUV) photon energies (10.49 9.71, and 9.34 eV, Figure 4) were selected to enable isomer-specific detection of products formed via radical–radical recombination following low-dose irradiation of formamide–methanol ices.

**Figure 3:**
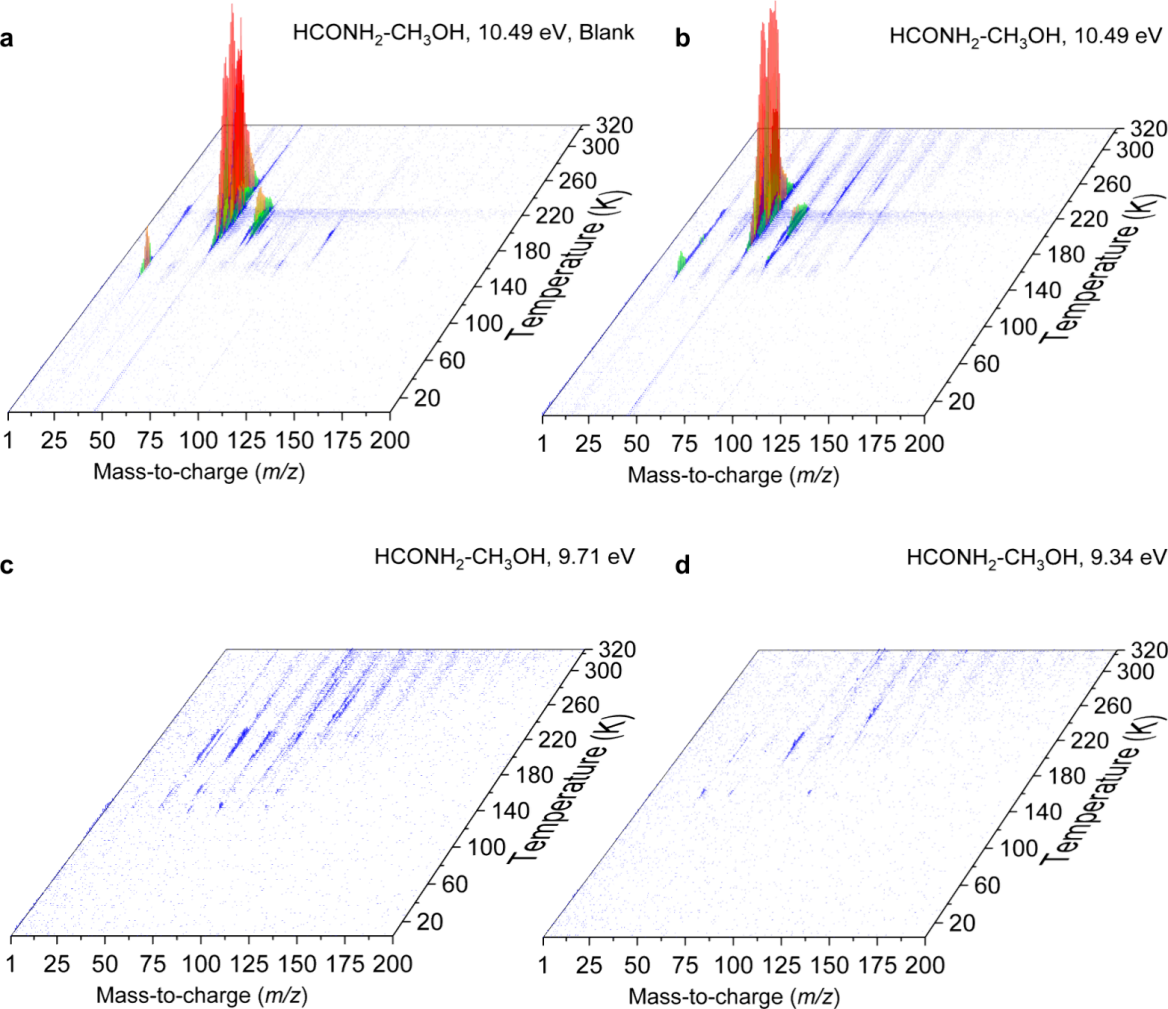
PI-ReTOF-MS data collected during the TPD of formamide–methanol ices exposed to doses of 0.40 ± 0.05 eV molecule^–1^. Panel (a) shows the unirradiated (blank) HCONH_2_/CH_3_OH ice recorded at 10.49 eV. Panels (b)–(d) display mass spectra of the irradiated ice recorded at photon energies of 10.49 eV (b), 9.71 eV (c), and 9.34 eV (d), respectively.

**Figure 4:**
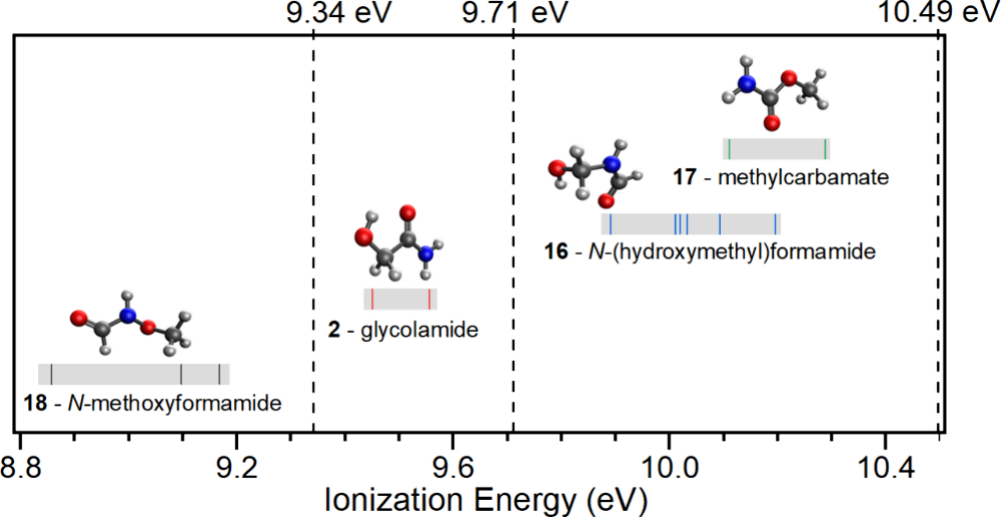
Computed adiabatic ionization energies (IEs) of isomers (solid lines) and ranges of their conformers (gray area) after error analysis (see Table S1). The VUV energies used for photoionization during the TPDs are indicated by dashed lines. Carbon atoms are shown in gray, hydrogen, in white, oxygen, in red, and nitrogen, in blue.

In Figure 4, the calculated ionization energies (IEs) for all conformers of isomers **2**, **16**, **17**, and **18** are plotted against the vacuum ultraviolet (VUV) photon energies used in this study; the vertical solid lines denote the IE of individual conformers, while the gray rectangles represent the full IE range of the conformers for each isomer, including error margins. The vertical dashed lines indicate the specific VUV photon energies employed in the experiments. An additional experiment at 8.8 eV was planned to distinguish between the signals of **2** and **18**, but this experiment was not needed as **18** was not detected in the 9.34 eV experiment (see Figure 5).

**Figure 5:**
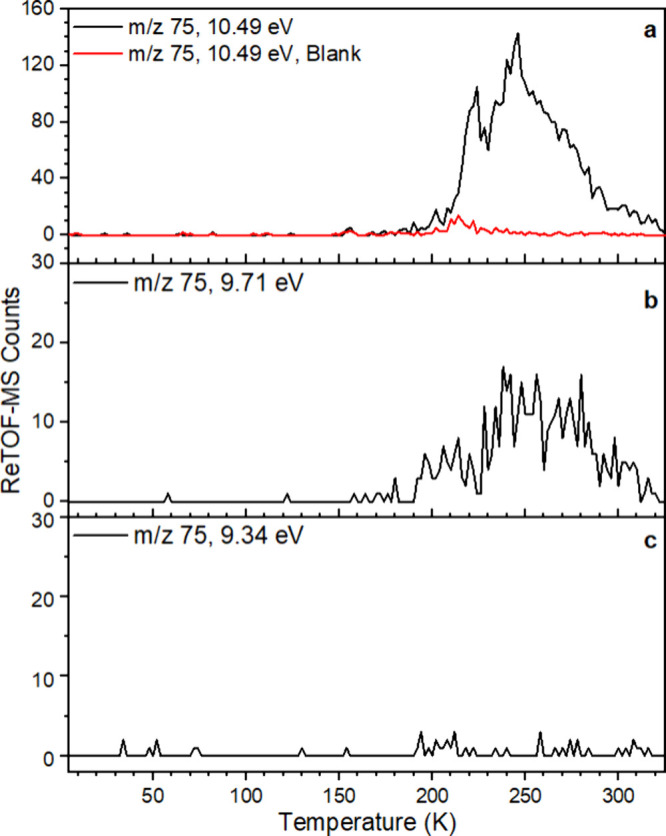
Sublimation profiles of selected mass-to-charge (*m*/*z*) signals recorded during the TPD. (a) *m*/*z* = 75 at 10.49 eV for the blank (nonirradiated) experiment (red trace) and the irradiated sample (black trace). (b) *m*/*z* = 75 at 9.71 eV; at this photon energy, only isomers **2** and **18** are expected to be ionized. (c) *m*/*z* = 75 at 9.34 eV; at this energy, only isomer **18** is expected to be ionized.

The very first experiment exploited a photon energy of 10.49 eV. At this energy, all four C_2_H_5_NO_2_ isomers (**2**, **16**, **17**, and **18**) formed through irradiation of formamide–methanol ices can be efficiently ionized and therefore are detectable via their ion counts by PI-ReTOF mass spectrometry. The corresponding result is shown in Figure 5a, where the black trace at *m*/*z* = 75 (10.49 eV) displays the signal from these isomers in comparison with the *m*/*z* = 75 signal from the blank experiment (Figure 5a, red trace). To refine the analysis, a second experiment was conducted under identical conditions, except that the photon energy was reduced to 9.71 eV. Based on the calculated IEs, only isomers **2** and **18** are ionizable at this energy. Thus, any signal observed at *m*/*z* = 75 amu under these conditions must originate from one or both species. The outcome of the 9.71 eV experiment is displayed in Figure 5b.

To further discriminate between isomers **2** and **18**, an additional experiment was performed at 9.34 eV, a photon energy below the IE of **2** (glycolamide) but still sufficient to ionize **18** (*N-methoxyformamide*). However, no signal was observed at *m*/*z* = 75 in this experiment (Figure 5c), indicating that **18** is not efficiently formed in this system. This absence implies that the signal detected at 9.71 eV (Figure 5b) originates solely from **2**, as glycolamide is the only remaining C_2_H_5_NO_2_ isomer ionizable at that energy.

To strengthen the confirmation of glycolamide detection, an additional experiment was conducted at 10.49 eV using an isotopically labeled ice mixture composed of formamide-*N*,*N*-*d*_2_ and methanol-OD. Since the parent species are only partially deuterated, the recombination of different pairs of first-generation radicals from formamide-*N*,*N*-*d*_2_ and methanol-OD causes different mass shifts on the isomers of interest, as outlined in Figure S2, which shows that the recombination of carbamoyl-*N*,*N*-*d*_2_ and hydroxymethyl-OD radicals results in glycolamide-*d*_3_ (**2**) (IUPAC name: 2-(deuterioxy)-*N*,*N*-*dideuterio*acetamide) at *m*/*z* = 78 amu. Therefore, the *m*/*z* = 78 signal observed in the formamide-*N*,*N*-*d*_2_/methanol-OD experiment (Figure S3a, red trace) corresponds exclusively to glycolamide, the only C_2_H_5_NO_2_ isomer expected to undergo a mass shift to 78 amu under these conditions. Figure S3b compares the *m*/*z* = 75 signal from the standard formamide–methanol experiment (black trace) with the *m*/*z* = 77 signal from the deuterated system (red trace), confirming the formation of species **16** and/or **17**. Critically, no *m*/*z* = 78 signal was detected in the nondeuterated experiment (Figure S3c), further supporting glycolamide (2) as the source of this mass channel in the formamide-*N*,*N*-*d*_2_/methanol-OD experiment.

## Discussion

Having established clear evidence for the formation of glycolamide (NH_2_C­(O)­CH_2_OH, **2**) as well as of *N*-(*hydroxymethyl*)­formamide (HCONHCH_2_OH, **16**) and/or methyl carbamate (NH_2_COOCH_3_, **17**) in formamide–methanol ices processed by electrons as proxies of secondary electrons generated by GCRs as they pass through astrophysical ices, we proceeded to analyze the reaction mechanisms leading to their synthesis.

The reaction sequence can be initiated by the cleavage of a carbon–hydrogen bond in formamide (**12**), producing the carbamoyl radical (ĊONH_2_, **10**) via reaction 1, an endoergic process with an energy requirement of 386.9 ± 0.7 kJ mol^–1^.^60^ Concurrently, a hydroxymethyl radical (ĊH_2_OH, **11**) is formed by the cleavage of a carbon–hydrogen bond in methanol through reaction 2, which is endoergic by 395.8 ± 0.3 kJ mol^–1^.^60^ This energy is supplied by the energetic electrons generated via interaction with GCRs. Subsequent radical–radical recombination between carbamoyl (**10**) and hydroxymethyl (**11**) radicals yields glycolamide through reaction 3, an exoergic process on the order of 350 kJ mol^–1^ (at 298.15 K) based on estimations using data from the *Active Thermochemical Tables* (ATcT) for the radicals^61^ and data from the literature for glycolamide:^62^

HCONH2(X1A′)→ĊONH2(X2A′)+Ḣ(S2) 1

CH3OH(X1A′)→ĊH2OH(X2A′)+Ḣ(S2) 2

$$Ċ{\mathrm{ONH}}_{2}\left(X^{2}A^{\prime }\right)+ĊH_{2}\mathrm{OH}\left(X^{2}A^{\prime }\right)\to {\mathrm{NH}}_{2}{\mathrm{COCH}}_{2}\mathrm{OH}\left(X^{1}A^{\prime }\right)$$ 3

Similarly, the homolytic cleavage of a nitrogen–hydrogen bond in formamide produces the formamido radical (HCOṄH, **14**), reaction 4, which is endoergic by 471 ± 1 kJ mol^–1^.^60^ Finally, the cleavage of the oxygen–hydrogen bond in methanol results in the methoxy radical (CH_3_Ȯ, **15**), an endoergic reaction by 434.9 ± 0.2 kJ mol^–1^ (reaction 5):^60^

HCONH2(X1A′)→HCOṄH(X2A′)+Ḣ(S2) 4

CH3OH(X1A′)→CH3Ȯ(X2E)+Ḣ(S2) 5

Consequently, the radical–radical recombination of **11** and **14** forms *N*-(*hydroxymethyl*)­formamide (HCONHCH_2_OH, **16**) through reaction 6; the recombination of **10** and **15** produces methyl carbamate (NH_2_COOCH_3_, **17**) via reaction 7, and the recombination of **14** and **15** produces *N-methoxyformamide* (CH_3_ONHCOH, **18**) through reaction 8:

$$ĊH_{2}\mathrm{OH}\left(X^{2}A^{\prime }\right)+\mathrm{HCO}ṄH\left(X^{2}A^{\prime }\right)\to {\mathrm{HCONHCH}}_{2}\mathrm{OH}\left(X^{1}A^{\prime }\right)$$ 6

$$Ċ{\mathrm{ONH}}_{2}\left(X^{2}A^{\prime }\right)+{\mathrm{CH}}_{3}Ȯ\left(X^{2}E\right)\to {\mathrm{NH}}_{2}{\mathrm{COOCH}}_{3}\left(X^{1}A^{\prime }\right)$$ 7

$$\mathrm{HCO}ṄH\left(X^{2}A^{\prime }\right)+{\mathrm{CH}}_{3}Ȯ\left(X^{2}E\right)\to {\mathrm{CH}}_{3}\mathrm{ONHCOH}\left(X^{1}A^{\prime }\right)$$ 8

However, we have found no evidence of *N-methoxyformamide* (**18**) in our experiments, suggesting that the formation of the nitrogen-centered formamido radical (HCOṄH, **14**) is disfavored relative to the carbon-centered carbamoyl radical (ĊONH_2_, **10**). This interpretation is supported by high-level calculations, which show that the C-centered carbamoyl isomer is significantly more stable than the N-centered (π) HCOṄH radical by 86.9 kJ mol^–1^.^63^ Therefore, it is possible that the signal from *m*/*z* = 77 from the isotopically labeled experiment (Figure S3) is exclusively from methyl carbamate (NH_2_COOCH_3_, **17**). Nevertheless, the data from this study alone do not allow definitive confirmation of this.

Recently, Perrero et al.^64^ proposed a computational alternative formation pathway for glycolamide on interstellar ice analogues, coupling formaldehyde (H_2_CO) and the carbamoyl (NH_2_CȮ) radical to form the intermediate NH_2_C­(O)­CH_2_Ȯ, which subsequently hydrogenates to yield glycolamide. Such a mechanism is predicted to become relevant at comparatively higher surface temperatures, where radical diffusion is enhanced. Under the low-temperature conditions used in these experiments, this pathway is unlikely to operate efficiently.

## Conclusions

Our combined experimental and computation study provided compelling evidence for the formation of glycolamide (NH_2_COCH_2_OH, **2**)–the first glycine (NH_2_CH_2_COOH, **1**) isomer detected in the interstellar medium^26^–in astrophysical model ices composed of formamide and methanol exposed to ionizing radiation (Figure 2). Although the composition of the experimental samples studied here (formamide/methanol ≈ 1:1) does not exactly reproduce the typical abundances in molecular clouds, this specific mixture was chosen as a proof of concept to elucidate formation of C_2_H_5_NO_2_ isomers, thereby enabling their detection via PI-ReTOF-MS, an approach that has been successfully employed in previous studies.^10,36,65^ The inclusion of additional common interstellar species, such as water, carbon monoxide, carbon dioxide, methane, and ammonia, would more closely reflect the composition of interstellar materials but would also substantially hinder the unambiguous identification of reaction products.^66^ Nonetheless, these results clearly demonstrate that glycolamide formation via the recombination of first-generation radicals produced from processed formamide and methanol is viable under astrophysical conditions. Because these reactions depend on encounters between radicals in favorable orientations, they remain feasible even in water-rich ices: although water enhances radical trapping and reduces diffusion, these effects do not necessarily alter the geometric and orientational factors that govern barrierless radical–radical branching.^67^ On the other hand, complementary experiments using ice mixtures in which water is the dominant component would provide valuable constraints for deriving realistic branching ratios and formation rates.

In conclusion, this study has found that glycolamide forms rapidly via barrierless recombination of first-generation radicals, carbamoyl and hydroxymethyl. These species were identified in the gas phase during temperature-programmed desorption (TPD) using tunable vacuum ultraviolet photoionization reflectron time-of-flight mass spectrometry (PI-ReTOF-MS) in combination with isotopic labeling experiments. The absence of glycine in the ISM may be partly explained by the scarcity of precursors that generate radicals capable of efficiently recombining barrierlessly to form glycine under low irradiation doses–a process supported by this study. Future experiments using methylamine (CH_3_NH_2_) and formic acid (HCOOH) mixtures will further test this hypothesis. Additional mechanisms, such as the nonenergetic route to glycine formation recently reported by Ioppolo et al.,^13^ were not investigated here. Such alternative routes warrant further examination to improve our understanding of the interstellar origins of prebiotic molecules. Furthermore, glycolamide may serve as a key astrochemical precursor, convertible into glycine (**1**), glycolic acid (**5**), lactic acid (**6**), alanine (**7**), and lactaldehyde (**8**) in deep space. C–O bond cleavage of glycolamide yields acetamide (**3**) and ethanolamine (**4**) with the latter oxidizing to glycine (Figure 1). These compounds are plausible precursors to complex amino acids, peptides, and sugars in the interstellar medium and, once formed in the solid phase, can be released during the early stages of planetary system formation, where they may be incorporated into planetesimals, seeding nascent planets with their first complex organic molecules.

## Laboratory Methods

All experiments were performed in an ultrahigh vacuum (UHV) chamber maintained at a base pressure of 5 × 10^–11^ Torr, achieved by ten magnetically levitated turbomolecular pumps backed by hydrocarbon-free dry scroll pumps. The substrates used were polished silver wafers (12.6 × 15.1 mm), mounted onto a cold head, and cooled to 5 K via a two-stage closed-cycle helium refrigerator. A new substrate was installed before each experiment. The reactants, formamide and methanol (Sigma-Aldrich, ≥99.5% purity and ≥99.9% purity, respectively), or their isotopically labeled analogs, formamide-*N*,*N*-*d*_2_ (HCOND_2_) and methanol-OD (both from CDN Isotopes, 99% atom D), were codeposited onto the silver substrate held at 5 K using two independent glass capillary arrays, which prevented any predeposition interaction between formamide and methanol. Both gases were introduced simultaneously at pressures of (4 ± 1) × 10^–9^ Torr each, for 20 ± 1 min, resulting in ice films with a thickness of 850 ± 50 nm. The thickness of each sample was monitored in real time via laser interferometry using a helium–neon laser (632.8 nm) and a photodiode connected to a picoammeter.^68^ The column densities of formamide and methanol were determined by FTIR based on nonoverlapping absorption bands at 1324 cm^–1^ (ν_7_ CN stretch, band strength of 0.85 × 10^–17^ cm)^52^ for formamide and at 1031 cm^–1^ (ν_8_ CO stretch, band strength of 1.07 × 10^–17^ cm)^54^ for methanol. After deposition, the ice mixture was irradiated with 5 keV electrons generated by an electron gun with an incident flux of 15 ± 2 nA for 15 min. Following irradiation, the ices were heated from 5 to 320 K at a constant rate of 1 K min^–1^ as part of the temperature-programmed desorption (TPD) process. Subliming molecules were photoionized by pulsed 30 Hz vacuum ultraviolet (VUV) photons, which were generated through resonant four wave mixing schemes inside a noble gas jet cell. The VUV photons were generated via sum-frequency generation (2ω_1_+ω_2_) for the 10.49 eV experiment and by difference-frequency generation (2ω_1_–ω_2_) for the 9.71 eV and 9.34 eV experiments. This was achieved by combining two Nd:YAG lasers (Spectra-Physics, Quanta Ray PRO 250-30 and 270-30), each coupled to a tunable dye laser (Sirah Lasertechnik, Cobra-Stretch). Detailed parameters for four-wave mixing exploited to generate the VUV photons are provided in Table S2. Additional details associated with the experimental apparatus and procedures are available in the Supporting Information.

### Safety

No unexpected or unusually high safety hazards were encountered in these experiments.

## Supplementary Material



## Notes

Experimental and computational details and methods including computed structures, energetics, and vibrational frequencies (PDF)

## †

Valongo Observatory, Federal University of Rio de Janeiro (OV-UFRJ), Rio de Janeiro-RJ, 20080-090, Brazil
