# Supplementary material for: Nonequilibrium Synthesis of Glycolamide (NH2COCH2OH), a Precursor to Amino Acids, on Interstellar Nanoparticles
Source: ACS Cent Sci. 2025 Dec 24;12(3):307–15. doi: 10.1021/acscentsci.5c01856 (PMC13022729; doi:10.1021/acscentsci.5c01856)
Supplement: Supplementary file 1 [file oc5c01856_si_001.pdf]

Supporting Information:
Non-Equilibrium Synthesis of Glycolamide (NH_2COCH_2OH), a Precursor to Amino Acids, on Interstellar Nanoparticles

Alexandre Bergantini[1,2,†], Jia Wang[1,2], Ivan Antonov[3], Evgenia A. Batrakova[3], Sergey O. Tuchin[3], Ralf I. Kaiser[1,2,*]

[1] *W. M. Keck Research Laboratory in Astrochemistry, University of Hawaii at Manoa, Honolulu, Hawaii 96822, United States*

[2] *Department of Chemistry, University of Hawaii at Manoa, Honolulu, Hawaii 96822, United States*

[3] *Samara National Research University, Samara, 443086 Russia*

†Present Address: *Valongo Observatory, Federal University of Rio de Janeiro (OV-UFRJ), Rio de Janeiro-RJ, 20080-090, Brazil*

[*] Corresponding Author: Ralf I. Kaiser, ralfk@hawaii.edu

MATERIALS AND METHODS

Experimental

All experiments were performed in a ultrahigh vacuum (UHV) chamber maintained at a base pressure of 5×10^{-11} Torr, achieved by ten magnetically levitated turbomolecular pumps (Osaka, TG1300MUCWB, TG420MCAB) backed by hydrocarbon-free dry scroll pumps (XDS35i, BOC Edwards). A polished silver substrate (12.6 × 15.1 mm) was mounted onto a cold head, cooled to 5 K via a two-stage closed-cycle helium refrigerator (Sumitomo Heavy Industries, RDK-415E). The cold head is capable of both vertical translation and free rotation through a doubly differentially pumped rotational feedthrough (Thermionics Vacuum Products, RNN-600/FA/MCO) and an adjustable bellows (McAllister, BLT86). The reactants, formamide ($HCONH_2$; Sigma-Aldrich, ≥99.5% purity) and methanol (Sigma Aldrich, ≥99.9% purity), or their isotopically labeled analogs, formamide-*N*,*N*-d_2 ($HCOND_2$; CDN Isotopes, 99% atom D) and methanol-OD (CH_3OD; CDN Isotopes, 99% atom D), were co-deposited onto the silver substrate at 5 K using

two independent glass capillary arrays to prevent pre-deposition interaction between formamide and methanol.

The gases were deposited at pressures of $(4 \pm 1) \times 10^{-9}$ Torr each, resulting in a total background pressure of $8 \pm 2 \times 10^{-9}$ Torr, for 20 ± 1 minutes. This process yielded an ice film $850 \pm 50\ nm$ of thickness. The film thickness was monitored in real time via laser interferometry using a helium-neon laser (632.8 nm) and a photodiode.[1] Prior to the deposition, the reactants were loaded into a glass vial interfaced to a UHV chamber and subjected to several freeze-pump-thaw cycles to remove residual atmospheric gases. The purified gases were then co-deposited onto the silver substrate at 5 K. An average refractive index of 1.35 ± 0.03 was used to determine the ice thickness of the $HCONH_2$-CH_3OH mixture, based on the individual refractive indices of formamide ice (n = 1.36)[2] and that of methanol ice (n = 1.33)[3]. The same average refractive index was applied to the isotopically labeled ice mixtures. A Fourier-transform infrared (FTIR) spectrometer (Thermo Fisher Scientific, Nicolet 6700) was used to analyze the ice after deposition over the spectral range of $6000 - 500$ cm^{-1} with a resolution of 2 cm^{-1}. FTIR spectra of the ice mixture were employed to determine the column densities of formamide and methanol based on non-overlapping absorption bands. The integrated infrared absorption of methanol at 1031 cm^{-1} (ν_8 CO stretch, $1.07 \times 10^{-17}\ cm$)[3] and formamide at 1324 cm^{-1} (ν_7 CN stretch, $0.85 \times 10^{-17}\ cm$)[2], were used to quantify each component. Integration was performed over the ranges 1079 to 987 cm^{-1} for methanol and 1366 to 1287 cm^{-1} for formamide, yielding a formamide-to-methanol ratio of (1.03 ± 0.05) to 1 prior to irradiation. Although the formamide-to-methanol ratio used in these experiments may not reflect typical abundances in molecular clouds, it was selected to maximize the yield of $C_2H_5NO_2$ isomers and thereby facilitate their detection, an approach that has been successfully employed in previous studies[4-6]. After deposition, the ice mixtures were irradiated with 5 keV electrons generated by an electron gun (SPECS, EQ 22/35, controller model EQ PU 22); the focused electron beam was rastered across the entire substrate area (1.6 cm^2) to ensure uniform exposure, with an incident flux of 15 ± 2 nA for 15 min, as measured by a Faraday cup positioned a few centimeters from the electron source. Prior to irradiation, a phosphor screen, fixed in the same plane, but a few centimeters above the substrate, was used to align the electron beam to ensure uniform coverage of the substrate. Monte Carlo simulations performed using the CASINO v2.42 software suite[7] estimated that the samples received a total dose of 0.40 ± 0.05 eV molecule^{-1}. This dose mimics the energy deposited by secondary electrons generated along the

ionization tracks of galactic cosmic rays (GCRs) in cold molecular clouds over lifetimes of up to 1×10^6 years[8]. The CASINO simulation also revealed that maximum penetration depth of 5 keV electrons in the formamide–methanol ice was $700 \pm 20\ nm$. Approximately 99% of the electron energy was deposited within the top $600 \pm 50\ nm$ of the sample, well below the total ice thickness of $850 \pm 50\ nm$, thus preventing interaction between the electrons and the underlying silver substrate. Following irradiation, the ices were heated from 5 K to 320 K at a constant rate of 1 K min^{-1} as part of the temperature-programmed desorption (TPD) process. Subliming molecules were photoionized by pulsed 30 Hz vacuum ultraviolet (VUV) photons, which were generated through resonant four wave mixing schemes inside a noble gas jet cell. The VUV photons were generated via sum-frequency generation ($2\omega_1 + \omega_2$; 10.49 eV) and difference-frequency generation (($2\omega_1 - \omega_2$; 9.71 eV, 9.34 eV) using two Nd:YAG lasers (Spectra-Physics, Quanta Ray PRO 250-30 and 270-30) coupled with two tunable dye lasers (Sirah Lasertechnik, Cobra-Stretch). Laser parameters are provided in Table S2. The resulting VUV radiation was spatially separated from residual laser beams using a biconvex lithium fluoride lens (Korth Kristalle, $R_1 = R_2 = 131$ mm) arranged in an off-axis geometry. The VUV beam passed approximately 2 mm above the ice surface, enabling selective photoionization of subliming molecules during the temperature-programmed desorption (TPD) process. The VUV photon flux was continuously monitored and recorded using a Faraday cup during the TPD and was employed to correct for variations in TPD profiles across individual experiments. Ions generated via VUV photoionization were analyzed using reflectron time-of-flight mass spectrometry and detected with a dual microchannel plate (MCP) detector (Jordan TOF Products). The ion signals were amplified using a preamplifier (Ortec, 9305), processed, and recorded by a multichannel scaler (FAST ComTec, MCS6A). Each recorded mass spectrum had a time resolution of 3.2 ns for ion arrival and an accumulation time of 2 minutes (corresponding to 3600 sweeps). A control experiment (blank) was conducted without electron irradiation at 10.49 eV for the $HCONH_2$–CH_3OH ice mixture. No signal was observed at *m/z* = 75 amu, aside from minor leakage caused by detector saturation from intense formamide desorption (see Fig. 5a, red trace). No unexpected or unusually high safety hazards were encountered in these experiments.

Computational Methods

Calculations were carried out using the Complete Basis Set Quadratic method with B3LYP geometry optimization *(CBS–QB3)* to obtain accurate energies for both the neutral and cationic states of each species, and are corrected by incorporating error[9]. The CBS-QB3 is a high-accuracy, composite chemistry model specifically designed for thermochemical calculations. Its reliability for computing ionization energies is well-established, as demonstrated in Montgomery et al.,[10] where it was shown to achieve a root-mean-square (rms) error of better than 0.05 eV for the experimental ionization energies included in the G2/97 set, providing high confidence in the accuracy of our computed values. This approach yields molecular parameters with high precision: bond lengths within 0.01–0.02 Å, bond angles within 1–2° and relative energies within 4–8 kJ mol^{-1}. All *ab initio* electronic structure calculations were performed using the GAUSSIAN 09 software package.[11] The adiabatic ionization energies (AIEs) were calculated as the difference in energy between the optimized neutral molecules and its corresponding cations at 0 K, including the zero-point vibrational energy (ZPVE). The equation used is:

$$AIE = \left[E_{elec(ion)} + E_{ZPVE(ion)}\right] - \left[E_{elec(neutral)} + E_{ZPVE(neutral)}\right] \qquad (1)$$

Where:

E_{elec} is the electronic energy.

E_{ZPVE} is the *is the zero-point vibrational energy.*

Four backbone isomers of the reaction products were identified: glycolamide (2), *N-(hydroxymethyl)*formamide (15), methylcarbamate (17), and *N-methoxyformamide* (18) (Figs. 2 and 4). For each backbone isomer, a range of conformers resulting from rotation around selected C–O, C–C, or C–N bonds was considered to ensure accurate determination of ionization potentials[6]. To ensure an exhaustive exploration of the conformer space, an initial geometry of each molecule, optimized at the B3LYP/6-31G* level, served as a template for generating all possible rotameric states. Systematic rotation about each conformer-active σ-bond (*n* in total) by 120° increments yielded a comprehensive set of 3^n initial structures. These geometries were subsequently optimized at the same B3LYP/6-31G* level. To identify and exclude redundant conformers (including optical isomers) from the resulting set, a custom filtering algorithm was employed. Each optimized structure was represented by a sorted list of interatomic distances corresponding to its third coordination sphere. This transformed each conformer into a point within a *k*-dimensional space defined by these selected distances. The Euclidean norm between all pairs

of points was calculated to construct a pairwise similarity metric. A histogram of these norms revealed a distinct bimodal distribution: values clustering near zero corresponded to identical or enantiomeric pairs, while a clear gap separated them from a population of larger norms representing pairs of dissimilar conformers. The threshold for uniqueness was objectively defined based on the position of the center of this gap. The geometries of these unique conformers, determined through the robust filtering procedure, subsequently served as the initial guesses for the accurate CBS-QB3 adiabatic ionization energy calculations detailed in the previous paragraph. This approach was validated through manual inspection and tested with different coordination spheres. The Cartesian coordinates, relative energies, ionization energies, and harmonic vibrational frequencies of the conformers of glycolamide, methylcarbamate, *N-(hydroxymethyl)*formamide, and *N-methoxyformamide* are compiled in Table S1.

SUPPORTING FIGURES

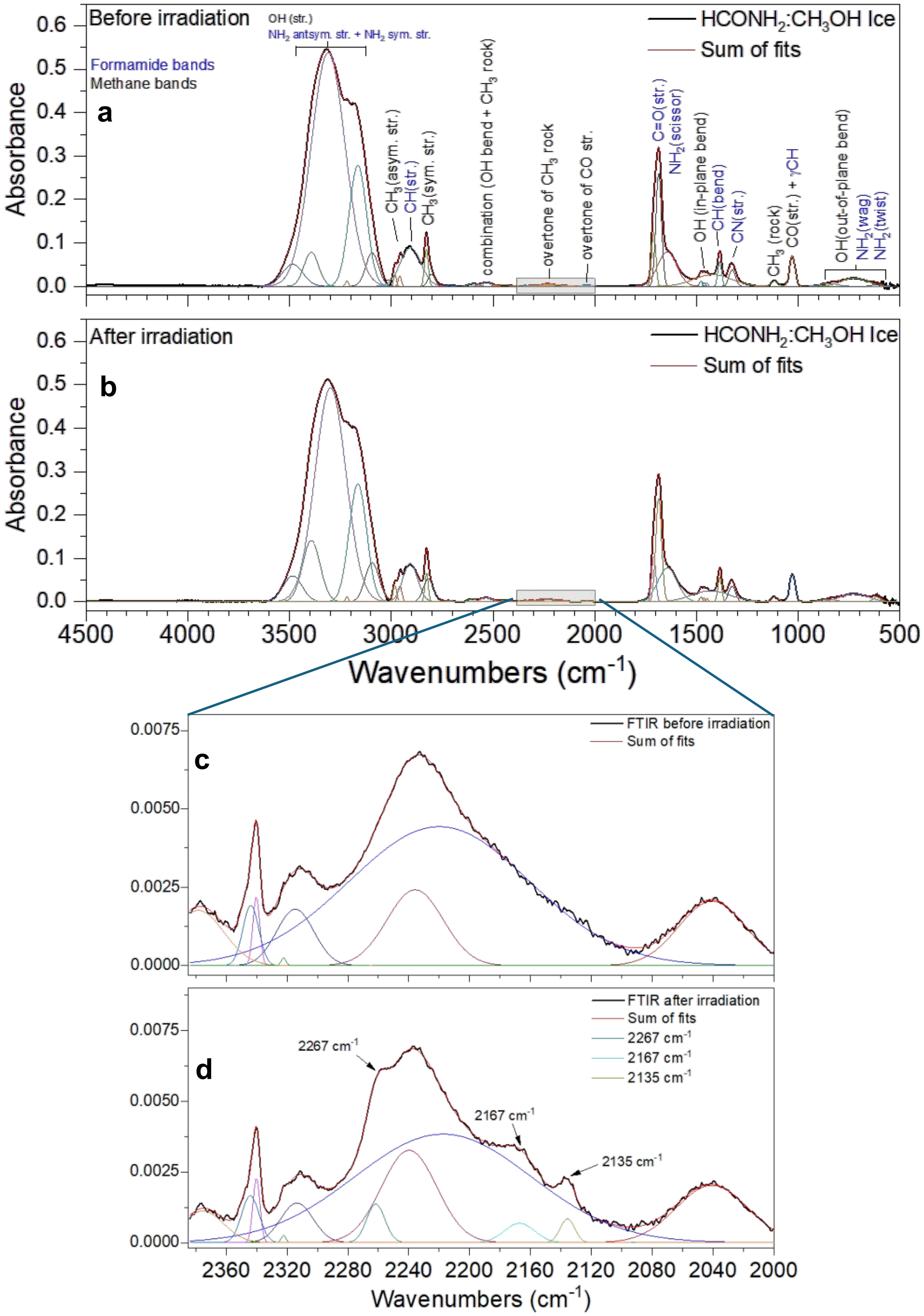

Figure S1: Infrared (FTIR) spectra of formamide-methanol ices recorded before (a) and after (b) irradiation with 5 keV electrons at 5 K. Panels (c) and (d) provide a magnified view of the 2385–2000 cm^{-1} region, highlighting the emergence of three new IR absorption bands following irradiation.

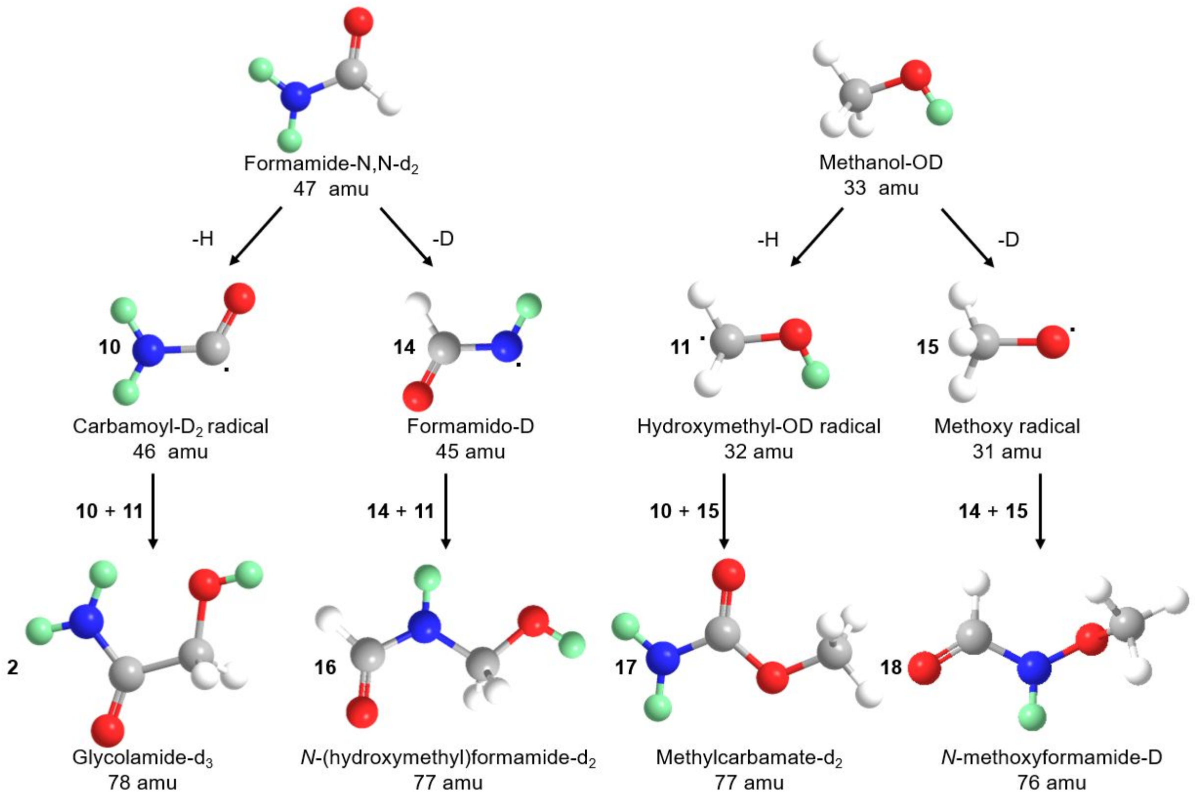

Figure S2: Reaction scheme leading to four first-generation $C_2H_{2\text{-}4}D_{1\text{-}3}O_2N$ (*m/z* = 76-78 amu) isomers synthesized in low-dose irradiation of formamide-N,N-d_2-methanol-OD ices at 5 K. Carbon atoms are shown in gray, hydrogen in white, oxygen in red, nitrogen in blue, and deuterium in light green.

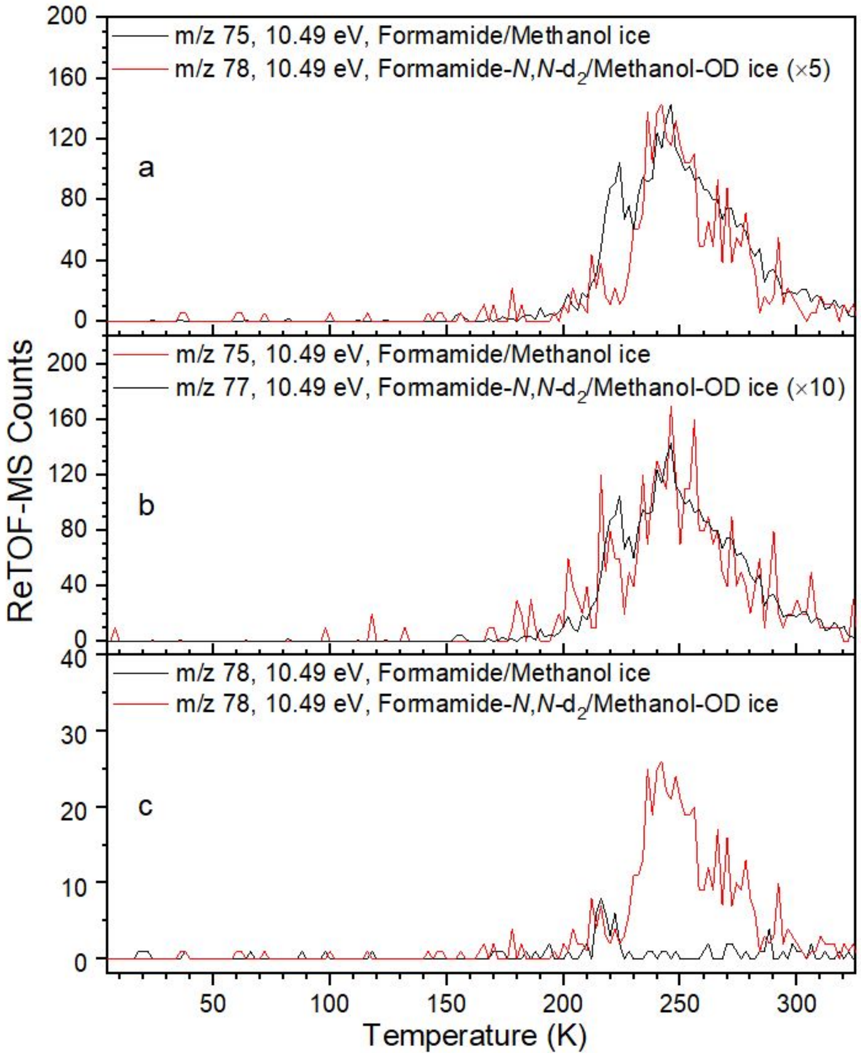

Figure S3: Comparison of PI-ReTOF-MS signals during TPD of irradiated samples in non-deuterated (black) versus partially-deuterated (red) experiments. (a) $C_2H_5NO_2$ isomers (black) vs. $C_2D_3H_2NO_2$ (red) which in this system exclusively corresponds to glycolamide glycolamide-d_3 ($ND_2C(=O)CH_2OD$). (b) $C_2H_5NO_2$ isomers (black) vs. $C_2D_2H_3NO_2$ (red) attributable to *N-(hydroxymethyl)*formamide-d_2 ($HCONDCH_2OD$) and/or methyl carbamate-d_2 ($ND_2C(=O)OCH_3$). (c) no signal at *m/z* = 78 amu was observed in the non-deuterated experiment (black) except for detector saturation from formamide sublimation; the red trace shows the TPD profile of glycolamide-d_3 ($ND_2C(=O)CH_2OD$) for comparison.

SUPPORTING TABLE

Table S1: Cartesian coordinates (Å), relative energies (ΔE), ionization energies (IE), and harmonic frequencies of conformers of glycolamide, methylcarbamate, *N*-(hydroxymethyl)formamide and *N*-methoxyformamide calculated at the composite CBS-QB3 level of theory. Carbon atoms are shown in gray, hydrogen in white, oxygen in red, and nitrogen in blue.

Glycolamide

Geometry	ΔE (kJ/mol)	IE (eV)	Frequencies (cm^{-1})
H 1.559400 0.913208 -0.000102<br>H 0.683246 -1.435779 0.885185<br>H 0.683258 -1.435654 -0.885378<br>H -2.549791 0.074012 -0.000159<br>H -1.803047 -1.488766 -0.000309<br>C 0.725549 -0.780346 -0.000041<br>C -0.507949 0.128934 -0.000003<br>N -1.714389 -0.487602 0.000117<br>O 1.884644 -0.003817 0.000055<br>O -0.369387 1.340650 -0.000029<br>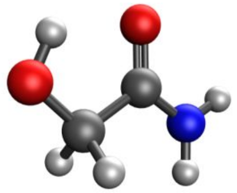	0.00000	9.45052	8.412160000000000082e+01 1.581136999999999944e+02 3.011102000000000203e+02<br>4.465962999999999852e+02 4.468842000000000212e+02 5.039187000000000012e+02<br>6.423174000000000206e+02 6.479652999999999565e+02 8.178854999999999791e+02<br>1.039310199999999895e+03 1.091884600000000091e+03 1.139471999999999980e+03<br>1.247579199999999901e+03 1.296977000000000089e+03 1.365782500000000027e+03<br>1.478480399999999918e+03 1.510633100000000013e+03 1.622265599999999949e+03<br>1.791876899999999978e+03 2.971511300000000119e+03 2.989730100000000220e+03<br>3.604808599999999842e+03 3.647577900000000227e+03 3.737734199999999873e+03<br><br>1.179809000000000054e+02 3.000599000000000274e+02 4.424848000000000070e+02<br>4.696970999999999776e+02 6.303515999999999622e+02 6.381050000000000182e+02<br>7.100484999999999900e+02 7.768682000000000016e+02 8.308993000000000393e+02<br>8.536322999999999865e+02 1.090948000000000093e+03 1.100068099999999959e+03<br>1.154184700000000021e+03 1.253560500000000047e+03 1.317294100000000071e+03<br>1.341129100000000108e+03 1.528353900000000067e+03 1.611376199999999926e+03<br>1.754689200000000028e+03 2.938297399999999925e+03 2.963181500000000142e+03<br>3.480287899999999809e+03 3.532819399999999860e+03 3.652320799999999963e+03
H -1.965636 0.326131 0.936978<br>H -0.799236 -1.207352 -1.106839<br>H -0.770705 -1.568640 0.618131<br>H 1.471740 1.779111 -0.072509<br>H -0.264260 1.728382 -0.235762<br>C -0.733626 -0.752517 -0.112908<br>C 0.646069 -0.089309 -0.003866<br>N 0.605096 1.270248 -0.017058<br>O -1.832926 0.139883 0.002175<br>O 1.660147 -0.752185 0.082830<br>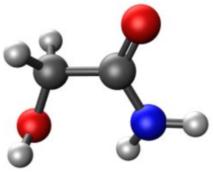	4.59200	9.55536	1.057304000000000030e+02 2.688394999999999868e+02 2.920776000000000181e+02<br>4.048883000000000152e+02 4.879313000000000216e+02 5.222030999999999494e+02<br>5.778605999999999767e+02 6.846420000000000528e+02 8.566960000000000264e+02<br>9.781909000000000560e+02 1.075351799999999912e+03 1.094965300000000070e+03<br>1.196352900000000091e+03 1.354149599999999964e+03 1.365373599999999897e+03<br>1.393653800000000047e+03 1.472623700000000099e+03 1.592130100000000084e+03<br>1.812376600000000053e+03 3.041690500000000156e+03 3.092107500000000073e+03<br>3.592544100000000071e+03 3.731152000000000044e+03 3.824121099999999842e+03<br><br>1.140575000000000045e+02 2.832529999999999859e+02 3.822497999999999934e+02<br>4.325892000000000053e+02 4.977427000000000135e+02 5.448578999999999724e+02<br>6.004035000000000082e+02 6.090945000000000391e+02 7.021298000000000457e+02<br>9.479157999999999902e+02 1.109714099999999917e+03 1.141381399999999985e+03<br>1.178396700000000010e+03 1.245142700000000104e+03 1.352382900000000063e+03<br>1.377769399999999905e+03 1.482693500000000085e+03 1.608889799999999923e+03<br>1.744937899999999900e+03 3.056415399999999863e+03 3.167176899999999932e+03<br>3.531524800000000141e+03 3.655015499999999975e+03 3.736165899999999965e+03

Methylcarbamate

Geometry	Energy (kJ/mol)	IE (eV)	Frequencies
H 2.528200 -0.039618 0.000045<br>H 1.671368 -1.558626 0.000012<br>H -2.564048 -0.910502 0.000033<br>H -1.974983 0.528421 -0.887780<br>H -1.974946 0.528433 0.887802<br>C 0.497414 0.142657 -0.000038<br>C -1.845889 -0.092963 0.000013<br>N 1.666382 -0.554338 0.000013<br>O 0.397222 1.347458 0.000005<br>O -0.554650 -0.718195 -0.000012<br>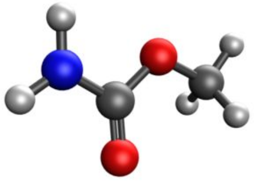	0.00000	10.2874	-2.024724999999999966e+02 9.778220000000000312e+01 1.647864999999999895e+02<br>2.923881000000000085e+02 4.770307999999999993e+02 5.359442999999999984e+02<br>6.688699000000000296e+02 7.824188000000000329e+02 8.662999999999999545e+02<br>1.086777000000000044e+03 1.114118099999999913e+03 1.177314399999999978e+03<br>1.213828099999999949e+03 1.358109799999999950e+03 1.478401399999999967e+03<br>1.481553200000000061e+03 1.506247200000000021e+03 1.612045599999999922e+03<br>1.826278299999999945e+03 3.045829200000000128e+03 3.114767899999999827e+03<br>3.149808599999999842e+03 3.628354400000000169e+03 3.764137200000000121e+03<br><br>8.686459999999999582e+01 1.752233999999999980e+02 2.290723000000000127e+02<br>3.001850000000000023e+02 4.733826000000000249e+02 5.792223999999999933e+02<br>6.068405999999999949e+02 7.139089000000000169e+02 8.410807999999999538e+02<br>1.027964999999999918e+03 1.096852900000000091e+03 1.161627799999999979e+03<br>1.193423000000000002e+03 1.222251800000000003e+03 1.451042200000000093e+03<br>1.473869500000000016e+03 1.486723999999999933e+03 1.590966400000000021e+03<br>1.635098799999999983e+03 3.079620300000000043e+03 3.181875900000000001e+03<br>3.216871599999999944e+03 3.466858400000000074e+03 3.592274399999999787e+03
H -0.200960 1.781780 -0.304773<br>H 1.486696 1.691402 -0.060304<br>H -2.606890 -0.740575 0.092937<br>H -1.977620 0.620420 -0.851421<br>H -1.831273 0.621463 0.936689<br>C 0.613565 -0.118021 -0.009712<br>C -1.810988 -0.000405 0.037198<br>N 0.583319 1.258981 0.048072<br>O 1.625179 -0.767162 0.020163<br>O -0.596259 -0.742437 -0.059482<br>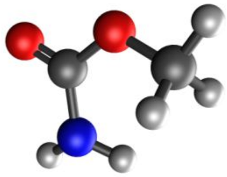	33.70092	10.1095	1.148769999999999953e+02 2.023179000000000087e+02 3.253711000000000126e+02<br>3.659010999999999854e+02 5.281509999999999536e+02 5.442948000000000093e+02<br>5.803345000000000482e+02 7.739337000000000444e+02 8.578714999999999691e+02<br>1.082029700000000048e+03 1.123687500000000000e+03 1.166179799999999886e+03<br>1.204817000000000007e+03 1.325854700000000093e+03 1.484495499999999993e+03<br>1.497551500000000033e+03 1.504868699999999990e+03 1.632818099999999959e+03<br>1.845656300000000101e+03 3.001167800000000170e+03 3.072093199999999797e+03<br>3.138347200000000157e+03 3.596500900000000001e+03 3.709710300000000188e+03<br><br>1.342070999999999970e+02 1.460030999999999892e+02 2.384765999999999906e+02<br>2.809617999999999824e+02 5.231643000000000256e+02 5.514738999999999578e+02<br>6.116233999999999469e+02 7.031834999999999809e+02 8.550725999999999658e+02<br>1.068098899999999958e+03 1.071616600000000062e+03 1.155384099999999989e+03<br>1.193921399999999949e+03 1.336833399999999983e+03 1.443491299999999910e+03<br>1.471941100000000006e+03 1.493755499999999984e+03 1.602641599999999926e+03<br>1.642889300000000048e+03 3.063186000000000149e+03 3.163112799999999879e+03<br>3.202336899999999787e+03 3.500946600000000217e+03 3.614539600000000064e+03

N-(hydroxymethyl)formamide

Geometry	Energy (kJ/mol)	IE (eV)	Frequencies
H 2.142562 0.777521 -0.625313<br>H 0.100487 1.829089 -0.621945<br>H -1.838890 1.120853 0.450830<br>H -0.860132 0.008197 1.439658<br>H -0.882726 -1.399465 -0.307737<br>C 1.294445 0.236145 -0.167952<br>C -1.061570 0.358347 0.422902<br>N 0.161410 0.983712 -0.074357<br>O 1.379868 -0.912353 0.224564<br>O -1.528421 -0.686288 -0.392650<br>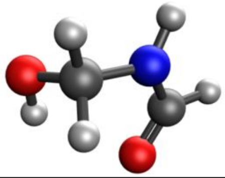	0.00000	10.1947	9.549280000000000257e+01 2.352524999999999977e+02 2.975366000000000213e+02<br>4.866361999999999739e+02 5.041974000000000160e+02 5.745789999999999509e+02<br>7.913929000000000542e+02 9.343790000000000191e+02 1.028812100000000100e+03<br>1.056704799999999977e+03 1.077698399999999992e+03 1.182637600000000020e+03<br>1.284281999999999925e+03 1.410680699999999888e+03 1.411830699999999979e+03<br>1.445471399999999903e+03 1.499041300000000092e+03 1.513328999999999951e+03<br>1.770926799999999957e+03 2.951151699999999892e+03 3.042281300000000101e+03<br>3.123032499999999800e+03 3.615870699999999943e+03 3.783791000000000167e+03<br><br>1.182965999999999980e+02 1.680945000000000107e+02 3.340047000000000139e+02<br>4.621815000000000282e+02 4.833258999999999901e+02 6.734541000000000395e+02<br>7.337939999999999827e+02 9.152921999999999798e+02 9.694084000000000287e+02<br>1.005058599999999956e+03 1.129678799999999910e+03 1.173506499999999960e+03<br>1.217298600000000079e+03 1.265431700000000092e+03 1.323953099999999949e+03<br>1.375753200000000106e+03 1.425935899999999947e+03 1.441138799999999947e+03<br>1.516505499999999984e+03 2.776579799999999977e+03 3.026376999999999953e+03<br>3.101316499999999905e+03 3.535279199999999946e+03 3.736125399999999900e+03
H -1.036920 0.009033 -1.431572<br>H -0.229589 0.285445 1.392616<br>H 1.648783 1.402678 0.272283<br>H 1.057465 0.696524 -1.246651<br>H 1.728737 -1.339867 -0.306061<br>C -1.241279 -0.003946 -0.341787<br>C 1.186292 0.520649 -0.171688<br>N -0.128746 0.319525 0.386267<br>O -2.327653 -0.261219 0.121211<br>O 2.085485 -0.537619 0.090835<br>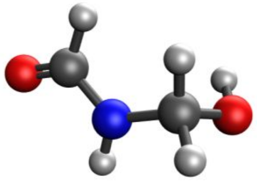	10.24733	10.0924	8.606910000000000593e+01 1.958265000000000100e+02 3.149184999999999945e+02<br>3.742318000000000211e+02 4.735663000000000125e+02 6.239886999999999944e+02<br>6.677777999999999565e+02 9.641353000000000293e+02 1.043642800000000079e+03<br>1.050987900000000081e+03 1.137396700000000010e+03 1.208203600000000051e+03<br>1.294862100000000055e+03 1.376608500000000049e+03 1.411736400000000003e+03<br>1.448914899999999989e+03 1.495431800000000067e+03 1.509939399999999978e+03<br>1.810274200000000064e+03 2.911668000000000120e+03 3.018358499999999822e+03<br>3.103541099999999915e+03 3.586298899999999776e+03 3.813835399999999936e+03<br><br>1.095213999999999999e+02 1.895379000000000076e+02 2.035397000000000105e+02<br>3.419936999999999898e+02 4.744187999999999761e+02 5.759388999999999896e+02<br>7.635311000000000377e+02 8.815239000000000260e+02 9.696352000000000544e+02<br>1.000515300000000025e+03 1.090986599999999953e+03 1.190536100000000033e+03<br>1.225640599999999949e+03 1.292281600000000026e+03 1.371677799999999934e+03<br>1.413495300000000043e+03 1.438340300000000070e+03 1.450424400000000105e+03<br>1.500089600000000019e+03 3.020219200000000001e+03 3.029827099999999973e+03<br>3.142072099999999864e+03 3.490068600000000060e+03 3.760478700000000117e+03
H -0.911127 0.647573 1.266388<br>H -0.320997 -0.973195 -1.122067<br>H 1.722158 -1.402016 -0.013508<br>H 1.178438 -0.430044 1.368144<br>H 2.048297 0.639750 -1.078292<br>C -1.186794 0.161373 0.311149<br>C 1.209409 -0.479503 0.279230<br>N -0.142449 -0.549513 -0.221058<br>O -2.288762 0.239354 -0.179143<br>O 1.931848 0.669809 -0.122799<br>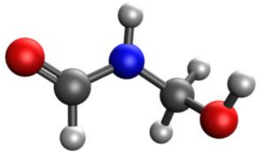	12.43174	9.89079	9.535819999999999652e+01 1.559290000000000020e+02 2.850543000000000120e+02<br>3.549707000000000221e+02 4.855530999999999722e+02 6.317338999999999487e+02<br>6.496331999999999880e+02 9.747353000000000520e+02 1.029259000000000015e+03<br>1.043149100000000089e+03 1.096667599999999993e+03 1.215188100000000077e+03<br>1.294569300000000112e+03 1.391673800000000028e+03 1.404099099999999908e+03<br>1.444213700000000017e+03 1.490037800000000061e+03 1.502592799999999897e+03<br>1.810095199999999977e+03 2.938006300000000010e+03 3.030117499999999836e+03<br>3.108634000000000015e+03 3.586880099999999857e+03 3.819901100000000042e+03<br><br>7.787980000000000302e+01 2.109276999999999873e+02 2.528427000000000078e+02<br>3.455939000000000192e+02 4.657850000000000250e+02 6.819543999999999642e+02<br>7.244030999999999949e+02 9.065017000000000280e+02 9.618995999999999640e+02<br>1.032888400000000047e+03 1.113590400000000045e+03 1.184431800000000067e+03<br>1.212317900000000009e+03 1.292377300000000105e+03 1.343197799999999916e+03<br>1.400526399999999967e+03 1.427269199999999955e+03 1.512630499999999984e+03<br>1.565958000000000084e+03 2.892699000000000069e+03 3.028496900000000096e+03<br>3.103475300000000061e+03 3.535509500000000116e+03 3.794709199999999782e+03
H 2.003400 1.077927 -0.672272<br>H -0.117237 1.723459 -0.238001<br>H -1.486881 0.483792 1.373814<br>H -0.493870 -0.946825 0.944065<br>H -2.523519 -0.869496 -0.226728<br>C 1.359402 0.308651 -0.205735<br>C -0.952112 -0.030018 0.564782<br>N 0.121015 0.805184 0.106191<br>O 1.741122 -0.816341 0.021945<br>O -1.825214 -0.280777 -0.531758	13.96241	10.0184	6.488679999999999382e+01 1.808494000000000028e+02 2.340654999999999859e+02<br>2.985903000000000134e+02 5.259348999999999705e+02 5.681706000000000358e+02<br>7.605796000000000276e+02 9.660493999999999915e+02 1.029684600000000046e+03<br>1.044403499999999894e+03 1.129530999999999949e+03 1.182917500000000018e+03<br>1.282112300000000005e+03 1.297652199999999993e+03 1.423806499999999915e+03<br>1.466646400000000085e+03 1.515685199999999895e+03 1.542601499999999987e+03<br>1.802478900000000067e+03 2.937469900000000052e+03 2.997124800000000050e+03<br>3.080682099999999991e+03 3.615746399999999994e+03 3.824412100000000009e+03<br><br>1.216680999999999955e+02 2.005925000000000011e+02 3.463802000000000021e+02<br>4.248315999999999804e+02 5.156165999999999485e+02 6.363016999999999825e+02<br>7.543638999999999442e+02 9.378944000000000187e+02 9.830807999999999538e+02<br>1.020070399999999950e+03 1.137786800000000085e+03 1.173599300000000085e+03<br>1.212966400000000021e+03 1.249171299999999974e+03 1.291546299999999974e+03<br>1.369459700000000112e+03 1.418390900000000101e+03 1.436909699999999930e+03

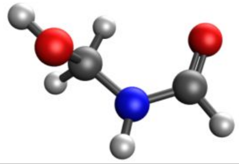			1.538452700000000050e+03 2.682724499999999807e+03 3.020364199999999983e+03<br>3.053529100000000199e+03 3.550815000000000055e+03 3.755283600000000206e+03
H -2.042729 1.037338 0.681680<br>H 0.045882 1.774600 0.267432<br>H 1.538428 0.669455 -1.262476<br>H 0.533473 -0.798783 -1.093632<br>H 2.326493 0.196585 0.828147<br>C -1.364669 0.297769 0.213148<br>C 0.972156 0.043405 -0.563868<br>N -0.126931 0.829342 -0.040051<br>O -1.708774 -0.829091 -0.056842<br>O 1.814029 -0.512364 0.427283<br>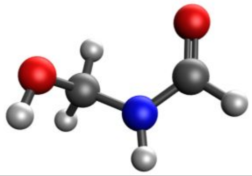	14.70543	10.0109	8.506180000000000518e+01 2.116687000000000012e+02 2.631703999999999724e+02<br>3.277826000000000022e+02 5.414650000000000318e+02 5.473961000000000467e+02<br>7.478238999999999805e+02 9.544352999999999838e+02 1.028442600000000084e+03<br>1.039504300000000057e+03 1.066845900000000029e+03 1.178358699999999999e+03<br>1.266967300000000023e+03 1.398960100000000011e+03 1.424143900000000031e+03<br>1.442786299999999983e+03 1.494989399999999932e+03 1.533160599999999931e+03<br>1.804918799999999919e+03 2.925972800000000007e+03 3.022293500000000222e+03<br>3.150582399999999780e+03 3.613864799999999832e+03 3.826599999999999909e+03<br><br>1.216398999999999972e+02 2.007177000000000078e+02 3.465221000000000231e+02<br>4.250002999999999815e+02 5.155883000000000038e+02 6.361378999999999451e+02<br>7.545163000000000011e+02 9.377747000000000526e+02 9.829426999999999452e+02<br>1.019639599999999973e+03 1.137258599999999888e+03 1.173724200000000110e+03<br>1.212754200000000083e+03 1.249134299999999939e+03 1.291477200000000039e+03<br>1.369393100000000004e+03 1.418112200000000030e+03 1.436565399999999954e+03<br>1.538500199999999950e+03 2.681469200000000001e+03 3.020392499999999927e+03<br>3.053789800000000014e+03 3.550488400000000183e+03 3.755330899999999929e+03
H 2.003400 1.077927 -0.672272<br>H -0.117237 1.723459 -0.238001<br>H -1.486881 0.483792 1.373814<br>H -0.493870 -0.946825 0.944065<br>H -2.523519 -0.869496 -0.226728<br>C 1.359402 0.308651 -0.205735<br>C -0.952112 -0.030018 0.564782<br>N 0.121015 0.805184 0.106191<br>O 1.741122 -0.816341 0.021945<br>O -1.825214 -0.280777 -0.531758<br>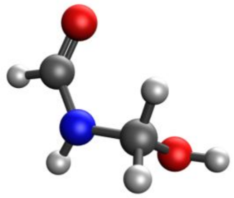	14.71330	10.0108	8.498980000000000246e+01 2.116799000000000035e+02 2.633240999999999872e+02<br>3.278820000000000050e+02 5.416431999999999789e+02 5.472997000000000298e+02<br>7.477913999999999533e+02 9.542845999999999549e+02 1.028497200000000021e+03<br>1.039622399999999971e+03 1.066826700000000073e+03 1.178367400000000089e+03<br>1.266901699999999892e+03 1.399149599999999964e+03 1.424202099999999973e+03<br>1.442676600000000008e+03 1.495063300000000027e+03 1.533162100000000009e+03<br>1.805012899999999945e+03 2.925754699999999957e+03 3.022179099999999835e+03<br>3.150427400000000034e+03 3.613841699999999946e+03 3.826756499999999960e+03<br><br>1.216402999999999963e+02 2.007181999999999960e+02 3.465233000000000061e+02<br>4.250015999999999963e+02 5.155911999999999580e+02 6.361326000000000249e+02<br>7.545205999999999449e+02 9.377736999999999625e+02 9.829426999999999452e+02<br>1.019640499999999975e+03 1.137267100000000028e+03 1.173721199999999953e+03<br>1.212755400000000009e+03 1.249126600000000053e+03 1.291475400000000036e+03<br>1.369387500000000045e+03 1.418112399999999980e+03 1.436568600000000060e+03<br>1.538495899999999892e+03 2.681470200000000204e+03 3.020389900000000125e+03<br>3.053799700000000030e+03 3.550495600000000195e+03 3.755327900000000227e+03
H -0.974564 -0.244520 -1.412187<br>H -0.247947 0.553906 1.328084<br>H 1.704993 1.392188 0.175917<br>H 1.099864 0.602393 -1.302208<br>H 2.807388 -0.573035 -0.074498<br>C -1.207605 -0.061461 -0.344927<br>C 1.186322 0.507564 -0.212226<br>N -0.136682 0.471165 0.325924<br>O -2.290197 -0.279806 0.143574<br>O 1.877039 -0.683408 0.149719<br>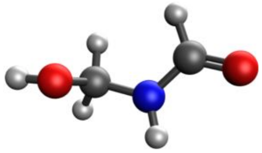	15.00998	10.0322	8.714570000000000505e+01 1.802459000000000060e+02 2.349251999999999896e+02<br>3.376494000000000142e+02 4.743768999999999778e+02 6.299415999999999940e+02<br>6.625139000000000351e+02 9.883487999999999829e+02 1.032653700000000072e+03<br>1.042940800000000081e+03 1.153855900000000020e+03 1.227588899999999967e+03<br>1.282316800000000057e+03 1.312093499999999949e+03 1.406422499999999900e+03<br>1.463810799999999972e+03 1.507224799999999959e+03 1.521208900000000085e+03<br>1.814880400000000009e+03 2.927155099999999948e+03 2.997878200000000106e+03<br>3.033391200000000026e+03 3.588863600000000133e+03 3.818120800000000145e+03<br><br>8.137980000000000302e+01 1.984227999999999952e+02 2.197343999999999937e+02<br>3.239291000000000054e+02 4.490240999999999758e+02 5.841761000000000195e+02<br>7.409651999999999816e+02 9.007563000000000102e+02 9.546930999999999585e+02<br>9.985009000000000015e+02 1.082074599999999919e+03 1.147874900000000025e+03<br>1.217001899999999978e+03 1.295088500000000067e+03 1.338276299999999992e+03<br>1.399810999999999922e+03 1.439067000000000007e+03 1.470442600000000084e+03<br>1.478003300000000081e+03 2.999275700000000143e+03 3.030312100000000100e+03<br>3.075109599999999773e+03 3.492147899999999936e+03 3.774209299999999985e+03

N-methoxyformamide

Geometry	Energy (kJ/mol)	IE (eV)	Frequencies
H -0.925386 1.393568 0.293508<br>H -0.278132 -1.461211 0.037004<br>H 2.359516 -0.788783 -0.837940<br>H 2.955801 0.488752 0.259846<br>H 1.850062 0.907903 -1.083556<br>C -1.219246 0.358410 0.044679<br>C 2.107903 0.151529 -0.336921<br>N -0.117903 -0.463042 -0.060188<br>O -2.349549 -0.011672 -0.161640<br>O 1.040989 -0.033149 0.599878<br>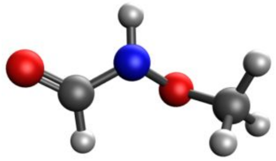	0.00000	9.09729	1.003511000000000024e+02 1.792274999999999920e+02 1.897599999999999909e+02<br>3.650828999999999951e+02 4.384395000000000095e+02 5.811658999999999651e+02<br>6.468339999999999463e+02 9.712105999999999995e+02 1.036220399999999927e+03<br>1.105166799999999967e+03 1.169822300000000041e+03 1.207578600000000051e+03<br>1.251703199999999924e+03 1.362359699999999975e+03 1.439278099999999995e+03<br>1.468392000000000053e+03 1.475280299999999897e+03 1.505176799999999957e+03<br>1.802354599999999891e+03 2.970769800000000032e+03 3.014058300000000145e+03<br>3.081097999999999956e+03 3.130147100000000137e+03 3.529402900000000045e+03<br><br>7.313110000000000355e+01 1.075892000000000053e+02 2.023233999999999924e+02<br>2.154274000000000058e+02 4.222948999999999842e+02 5.189815999999999576e+02<br>5.655675999999999704e+02 8.351743000000000166e+02 9.689881000000000313e+02<br>9.920707999999999629e+02 1.133451900000000023e+03 1.168023100000000113e+03<br>1.310015100000000075e+03 1.345150399999999991e+03 1.438450599999999895e+03<br>1.458225699999999961e+03 1.472038299999999936e+03 1.502784699999999930e+03<br>1.816048399999999901e+03 3.055470100000000002e+03 3.091150799999999890e+03<br>3.159475399999999809e+03 3.205614099999999780e+03 3.401790899999999965e+03
H 2.144627 0.874776 -0.529076<br>H 0.150472 1.896463 -0.109059<br>H -2.060703 -0.025105 -1.165597<br>H -2.532733 -0.849348 0.351388<br>H -1.095035 -1.408388 -0.548729<br>C 1.339689 0.212940 -0.148246<br>C -1.693845 -0.538650 -0.271521<br>N 0.141703 0.885467 -0.157278<br>O 1.499234 -0.944861 0.147619<br>O -0.933435 0.353310 0.554960<br>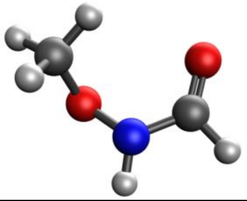	1.17622	9.16769	9.200759999999999650e+01 1.884927999999999884e+02 2.750493000000000166e+02<br>2.853201000000000249e+02 4.914390999999999963e+02 5.166648000000000138e+02<br>8.181343000000000529e+02 9.244968999999999824e+02 1.010672500000000014e+03<br>1.038735799999999927e+03 1.165691800000000057e+03 1.201898400000000038e+03<br>1.220535499999999956e+03 1.407663900000000012e+03 1.454738000000000056e+03<br>1.472608899999999949e+03 1.482161599999999908e+03 1.509281300000000101e+03<br>1.812987300000000005e+03 2.910591100000000097e+03 3.021033800000000156e+03<br>3.099160499999999956e+03 3.134180699999999888e+03 3.573839899999999943e+03<br><br>8.303780000000000427e+01 1.922504999999999882e+02 2.486989000000000090e+02<br>2.980561999999999898e+02 3.780570999999999913e+02 5.967605999999999540e+02<br>7.121921999999999571e+02 7.934741999999999962e+02 8.704367999999999483e+02<br>9.763152000000000044e+02 1.115289700000000039e+03 1.171931399999999940e+03<br>1.300698000000000093e+03 1.396848199999999906e+03 1.435119699999999966e+03<br>1.458622800000000097e+03 1.469929399999999987e+03 1.523898200000000088e+03<br>1.790398400000000038e+03 3.064524699999999939e+03 3.069310399999999845e+03<br>3.173521699999999782e+03 3.211951300000000174e+03 3.464169199999999819e+03
H -1.740952 -0.786832 0.000000<br>H 0.528404 1.134651 0.000000<br>H 2.569274 -1.682589 0.000000<br>H 2.414690 -0.149294 0.898602<br>H 2.414690 -0.149294 -0.898602<br>C -1.356097 0.247647 0.000000<br>C 2.104555 -0.697954 0.000000<br>N 0.000000 0.271849 0.000000<br>O -2.042788 1.247771 0.000000<br>O 0.708181 -0.943739 0.000000<br>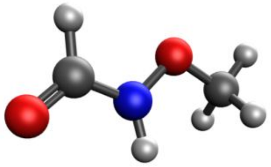	23.12278	8.85688	-3.732869000000000028e+02 -7.356380000000000052e+01 2.229961999999999875e+02<br>2.472944999999999993e+02 4.091084000000000174e+02 4.584196000000000026e+02<br>5.786136999999999944e+02 1.001630800000000022e+03 1.030431800000000067e+03<br>1.103098099999999931e+03 1.171395999999999958e+03 1.215279999999999973e+03<br>1.287150100000000066e+03 1.366680800000000090e+03 1.454750600000000077e+03<br>1.475074299999999994e+03 1.493362900000000081e+03 1.513087899999999991e+03<br>1.788292300000000068e+03 2.976187800000000152e+03 2.984972400000000107e+03<br>3.038245699999999943e+03 3.140107399999999870e+03 3.588596799999999803e+03<br><br>7.144240000000000634e+01 1.061795999999999935e+02 2.018862000000000023e+02<br>2.155514000000000010e+02 4.222386999999999944e+02 5.188464999999999918e+02<br>5.652464999999999691e+02 8.344846999999999753e+02 9.689461000000000013e+02<br>9.919279000000000224e+02 1.132743199999999888e+03 1.167164600000000064e+03<br>1.309447599999999966e+03 1.345424299999999903e+03 1.437855099999999993e+03<br>1.458036399999999958e+03 1.471726799999999912e+03 1.502083800000000110e+03<br>1.815400399999999991e+03 3.055579200000000128e+03 3.091204699999999775e+03<br>3.159869900000000143e+03 3.205526199999999790e+03 3.402316100000000006e+03

Table S2: Parameters for VUV generation ($2\omega_1 \pm \omega_2$).

	Photoionization energy	10.49 (eV)	9.71 (eV)	9.34 (eV)
	Wavelength (nm)	118.19	127.68	132.74
ω_1	Wavelength (nm) – Nd:YAG laser #1	354.66	532.00	532.00
	Wavelength (nm) – Dye laser #1	-	606.948	606.948
ω_2	Wavelength (nm) – Nd:YAG laser #2	-	354.66	354.66
	Wavelength (nm) – Dye laser #2	-	486.88	425.11
	Non-linear medium	Xe	Kr	Kr

References:

(1) Turner, A. M.; Abplanalp, M. J.; Chen, S. Y.; Chen, Y. T.; Chang, A. H. H.; Kaiser, R. I. A photoionization mass spectroscopic study on the formation of phosphanes in low temperature phosphine ices. *Physical Chemistry Chemical Physics* **2015**, *17* (41), 27281-27291. DOI: 10.1039/C5CP02835C.
(2) Brucato, J. R.; Baratta, G. A.; Strazzulla, G. An infrared study of pure and ion irradiated frozen formamide. *Astronomy & Astrophysics* **2006**, *455* (2), 395-399. DOI: 10.1051/0004-6361:20065095.
(3) Bouilloud, M.; Fray, N.; Bénilan, Y.; Cottin, H.; Gazeau, M.-C.; Jolly, A. Bibliographic review and new measurements of the infrared band strengths of pure molecules at 25 K: H_2O, CO_2, CO, CH_4, NH_3, CH_3OH, HCOOH and H_2CO. *Monthly Notices of the Royal Astronomical Society* **2015**, *451* (2), 2145-2160. DOI: 10.1093/mnras/stv1021.
(4) Bergantini, A.; Maksyutenko, P.; Kaiser, R. I. On the Formation of the C_2H_6O Isomers Ethanol (C_2H_5OH) and Dimethyl Ether (CH_3OCH_3) in Star-forming Regions. *The Astrophysical Journal* **2017**, *841* (96), 1-24. DOI: 10.3847/1538-4357/aa7062.
(5) Bergantini, A.; Góbi, S.; Abplanalp, M. J.; Kaiser, R. I. A Mechanistical Study on the Formation of Dimethyl Ether (CH3OCH3) and Ethanol (CH3CH2OH) in Methanol-containing Ices and Implications for the Chemistry of Star-forming Regions. *The Astrophysical Journal* **2018**, *852* (2), 1-13. DOI: 10.3847/1538-4357/aa9ce2.
(6) Wang, J.; Zhang, C.; Marks, J. H.; Evseev, M. M.; Kuznetsov, O. V.; Antonov, I. O.; Kaiser, R. I. Interstellar formation of lactaldehyde, a key intermediate in the methylglyoxal pathway. *Nature Communications* **2024**, *15* (1), 10189. DOI: 10.1038/s41467-024-54562-x.
(7) Drouin, D.; Couture, A. R.; Joly, D.; Tastet, X.; Aimez, V.; Gauvin, R. CASINO V2. 42—A Fast and Easy-to-use Modeling Tool for Scanning Electron Microscopy and Microanalysis Users. *Scanning* **2007**, *29* (3), 92-101. DOI: 10.1002/sca.20000.
(8) Yeghikyan, A. G. Irradiation of dust in molecular clouds. II. Doses produced by cosmic rays. *Astrophysics* **2011**, *54* (1), 87-99. DOI: 10.1007/s10511-011-9160-2.
(9) Montgomery Jr, J. A.; Frisch, M. J.; Ochterski, J. W.; Petersson, G. A. A complete basis set model chemistry. VI. Use of density functional geometries and frequencies. *The Journal of chemical physics* **1999**, *110* (6), 2822-2827.
(10) Montgomery Jr, J. A.; Frisch, M. J.; Ochterski, J. W.; Petersson, G. A. A complete basis set model chemistry. VII. Use of the minimum population localization method. *The Journal of Chemical Physics* **2000**, *112* (15), 6532-6542.
(11) Frisch, M. J., et al. gaussian 09, Revision d. 01, Gaussian. *Inc, Wallingford CT* **2013**, *201*.
